# Distinct mechanical behavior of HEK293 cells in adherent and suspended states

**DOI:** 10.7717/peerj.1131

**Published:** 2015-07-30

**Authors:** Seyed Mohammad Ali Haghparast, Takanori Kihara, Jun Miyake

**Affiliations:** 1 Department of Mechanical Science and Bioengineering, Graduate School of Engineering Science, Osaka University, Toyonaka, Osaka, Japan; 2 Department of Life and Environment Engineering, Faculty of Environmental Engineering, The University of Kitakyushu, Kitakyushu, Fukuoka, Japan

**Keywords:** Mechanical features, Atomic force microscopy, HEK293 cells, Suspended state, Adherent state, Actin cytoskeleton

## Abstract

The mechanical features of individual animal cells have been regarded as indicators of cell type and state. Previously, we investigated the surface mechanics of cancer and normal stromal cells in adherent and suspended states using atomic force microscopy. Cancer cells possessed specific mechanical and actin cytoskeleton features that were distinct from normal stromal cells in adherent and suspended states. In this paper, we report the unique mechanical and actin cytoskeletal features of human embryonic kidney HEK293 cells. Unlike normal stromal and cancer cells, the surface stiffness of adherent HEK293 cells was very low, but increased after cell detachment from the culture surface. Induced actin filament depolymerization revealed that the actin cytoskeleton was the underlying source of the stiffness in suspended HEK293 cells. The exclusive mechanical response of HEK293 cells to perturbation of the actin cytoskeleton resembled that of adherent cancer cells and suspended normal stromal cells. Thus, with respect to their special cell-surface mechanical features, HEK293 cells could be categorized into a new class distinct from normal stromal and cancer cells.

## Introduction

Investigation of the mechanical features provides specific information about the different states and types of animal cells. For example, mechanical features of mesenchymal stem cells are attributed to their diverse characteristics and states (Kihara et al., 2011; Maloney et al., 2010; Sugitate et al., 2009). In addition, malignant cancer cells exhibit lower stiffness than normal cells (Cross et al., 2007; Guck et al., 2005; Haghparast et al., 2013; Suresh et al., 2005); cortical stiffness changes during mitotic cell rounding (Kunda et al., 2008; Shimizu et al., 2012a); and the stiffness of the retinal epithelium changes during optic-cup morphogenesis (Eiraku et al., 2011). Such mechanical features and alterations are largely attributable to the actin cytoskeleton (Dai & Sheetz, 1995; Sugitate et al., 2009; Trickey, Vail & Guilak, 2004; Wang, 1998). Stress fibers are specific determinants of cellular mechanics (Lu et al., 2008); the actin cap and cortical actin have also been reported as promoters of cortical rigidity (Kihara et al., 2011; Kunda et al., 2008; Maddox & Burridge, 2003). Thus, analyzing the mechanical features of cells can reveal the characteristics of their underlying actin networks. It is also possible to characterize the sub-membrane actin networks in each cell type as indicators of surface stiffness.

There are several methods used to measure the mechanical features of cells, including micropipette aspiration (Evans & Yeung, 1989), optical stretching (Guck et al., 2000), and atomic force microscopy (AFM) (Radmacher et al., 1996). In particular, AFM can be used to investigate the mechanical properties of the cell surface with high sensitivity (∼1 pN) and spatial resolution (∼1 nm) under physiological cell culture conditions (Haga et al., 2000; Kihara et al., 2013; Matzke, Jacobson & Radmacher, 2001; Rotsch et al., 1997). Furthermore, by using a substrate coated with hydrophilic cell-anchoring molecules (Kato et al., 2003), AFM can be used to measure the stiffness of suspended leukocytes and trypsinized cells (Haghparast et al., 2013; Kagiwada et al., 2010; Shimizu et al., 2012a; Shimizu et al., 2012b). The substrate-adherent cells have anisotropic and heterogeneous actin cytoskeleton, but the suspended cells, which are removed from the substrate by trypsinization, exhibit an apparently isotropic actin cytoskeleton in the vicinity of plasma membrane (Shimizu et al., 2012a). In order to characterize these actin structures, it is required to measure the surface stiffness in both adhesion conditions. AFM measurement has the potential to detect the surface stiffness of any adherent and suspended cell types.

Previously, we reported the surface mechanics and actin cytoskeleton architecture of normal stromal cells i.e., mesenchymal stem cells and normal fibroblasts, and cancer cells i.e., HeLa and HT1080 cells in adherent and suspended states using AFM (Haghparast et al., 2013). Cancer cells possessed specific mechanical and actin cytoskeleton features that were distinct from those of normal stromal cells in adherent and suspended states (Haghparast et al., 2013). The regulatory mechanisms of F-actin structures were different between normal stromal and cancer cells, regardless of their adhesion state (Haghparast et al., 2013). Therefore, we assumed that cells could be categorized into two main groups with respect to their mechanical features: normal stromal cells and cancer cells (Haghparast et al., 2013). In this study, we examined the surface stiffness of embryonic kidney HEK293 cells in adherent and suspended states using the AFM indentation method. HEK293 cells are transformed cell line with adenovirus (Graham et al., 1977) and they show cancer-like behavior in tissue culture. We unexpectedly found that HEK293 cells belonged to a third group of cells with specific mechanical features that differed from those of normal stromal and cancer cells.

## Materials and Methods

### Materials

**Figure 1:**
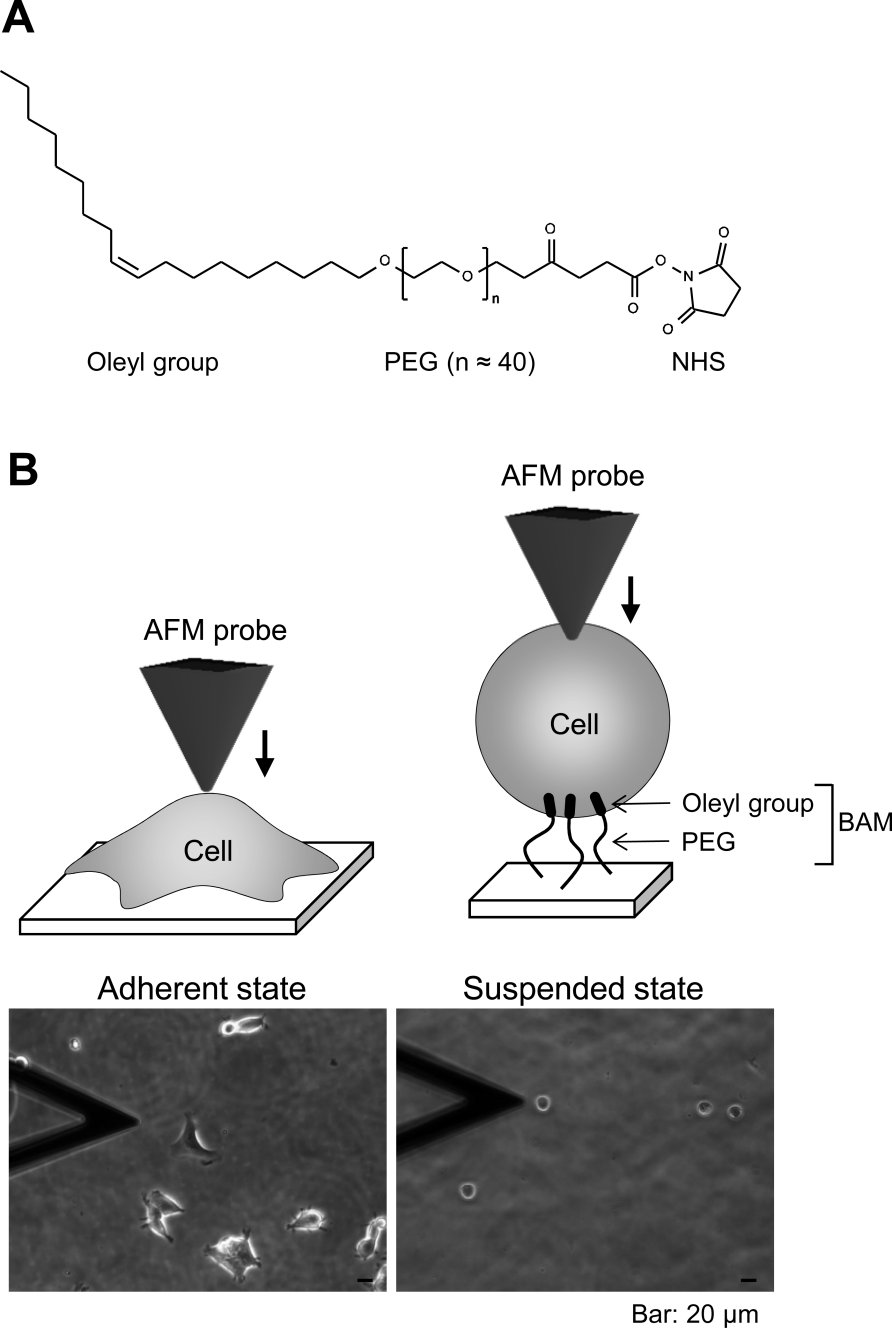
Atomic force microscopy (AFM) manipulation of cultured cells. (A) Chemical structure of the BAM molecule. It comprises an oleyl group, an NHS-reactive ester group, and a hydrophilic PEG linker. (B) Diagrams of AFM manipulation of the adhered or BAM-anchored suspended cells (upper images). Phase-contrast micrographs (lower images) of adherent and BAM-anchored suspended HEK293 cells (the deltoidal object on the left is the AFM cantilever).

The pyramidal probe (SN-AF01S-NT; spring constant: 0.02 N/m) was purchased from Seiko Instruments Inc. (Tokyo, Japan). Human embryonic kidney HEK293 cells were obtained from Health Science Research Resources Bank (Osaka, Japan). Cell anchoring molecule, BAM (SUNBRIGHT OE-020CS) (Fig. 1A), was purchased from NOF Corporation (Tokyo, Japan). F-actin labeling kit was purchased from AAT Bioquest, Inc. (Sunnyvale, California, USA). Other reagents were purchased from Sigma-Aldrich (St. Louis, Missouri, USA), Wako Pure Chemical Industries Ltd. (Osaka, Japan), or Life Technologies Japan Ltd. (Tokyo, Japan).

### Preparation of BAM-coated dishes

BAM-coated dishes were prepared as described previously (Haghparast et al., 2013). The BAM molecule contains an oleyl group at one end that anchors the suspended cells (Kato et al., 2003). BAM-anchored suspended cells do not move around like floating cells and cannot spread on the substrate like adherent cells on normal tissue culture surface (Shimizu et al., 2012a; Shimizu et al., 2012b). Thus, we can measure the stiffness of suspended cells by AFM using BAM-coated dishes (Haghparast et al., 2013). Tissue culture dishes were coated with 5% BSA in PBS for 1 h. The BSA layer associates with NHS group of BAM molecule and prevents any non-specific cell-substrate interaction. After washing with ultra-pure water, the surfaces were treated with 1 mM BAM molecules in PBS for 30 min. Then, the BAM-coated dishes were washed and dried.

### Cell culture and drug treatment

HEK293 cells were maintained in DMEM containing 10% FBS and antibiotics (penicillin and streptomaycin). The culture medium was replaced twice a week. For adherent and suspended states examination, the cells were treated with Y27632 (20 µM) or calyculin A (0.1 nM) for 12 h. For the suspended state, the cells were removed from the culture dish by treating with 0.25% trypsin-0.02% EDTA in PBS and plated on a BAM-coated dish for 30 min in complete culture medium, then washed with PBS to remove un-attached cells, and cultured for 12 h in drug-containing medium. The cells adhered to uncoated and BAM-coated culture dishes with or without the drug treatment were manipulated by AFM (Fig. 1B). Actin depolymerization was induced by treating the cells with 5 µM cytochalasin D for 2 h.

### Fluorescent image of the actin cytoskeleton

To visualize actin cytoskeleton microstructures, cultured cells in glass bottom dishes with or without BAM coating were fixed with 4% paraformaldehyde, permeabilized with 0.5% Triton X-100, and stained with the F-actin labeling kit. Serial sections of specimens (0.5 µm thick) were observed by confocal laser scanning microscopy (CLSM) (FV-1000; Olympus, Tokyo, Japan) using a 60× oil immersion lens (NA = 1.42). Serial images were superimposed using ImageJ software (NIH, Bethesda, Maryland, USA).

### AFM measurements

Single adherent and BAM-anchored suspended HEK293 cells in medium were manipulated by AFM (Nanowizard I; JPK Instruments AG, Berlin, Germany) at room temperature. Combining the optical microscope (IX-71; Olympus) and AFM allows the probe to be placed on a particular region of the cell surface. In this study, the AFM probe was indented the cell surface on the nuclear region with a loading force of up to 1 nN and velocity of 5 µm/s. The Young’s modulus of the cell was calculated using the Hertz model (Hertz, 1881). The force–distance curve for a region up to about 500 nm of cell surface indentation was fitted using JPK data processing software (JPK instruments AG, Berlin, Germany) as:

(1) \begin{eqnarray*} \displaystyle F=\frac{E}{1-{\nu }^{2}}\frac{\tan \alpha }{\sqrt{2}}{\delta }^{2},&&\displaystyle \end{eqnarray*}

where *F* = force, *δ* = depth of the probe indentation, *ν* = Poisson’s ratio (0.5), *α* = half-angle to the face of the pyramidal probe (20°), and *E* = Young’s modulus. More than 20 cells were used per experiment, and 25 points were examined on the surface of each cell. The median value was adopted as the Young’s modulus of each cell (Kihara et al., 2011). The logarithmic values of the Young’s modulus were used for the statistical analysis (Haghparast et al., 2013). Young’s moduli of the polystyrene tissue culture surface and the BAM coated surface were 1.02 × 10^7^ and 1.27 × 10^7^ Pa, respectively (Supplementary raw data of Young’s modulus of substrate surface). The range of Young’s moduli of cell surface was in order of about 10^2^ Pa. Thus, we were convinced that the surface stiffness of cultured cells could be measured by this method without affecting the surface rigidity of these culture substrates.

### Statistical analysis

The logarithmic Young’s modulus values for each group were compared by analysis of variance followed by Mann–Whitney *U* test. *p*-values of less than 0.01 were considered as statistically significant.

## Results

### Actin cytoskeleton structures of adherent and BAM-anchored suspended HEK293 cells

First, we observed the actin cytoskeleton of HEK293 cells in adherent and suspended states. The cells adhered to and weakly spread along the normal culture substrate (Fig. 1B). After cell detachment from the culture substrate by trypsinization, the suspended cells were seeded on a BAM-coated culture dish. The suspended cells were immobilized on the BAM surface, which prevents cell spreading, and maintained their round shape after 12 h (Fig. 1B).

**Figure 2:**
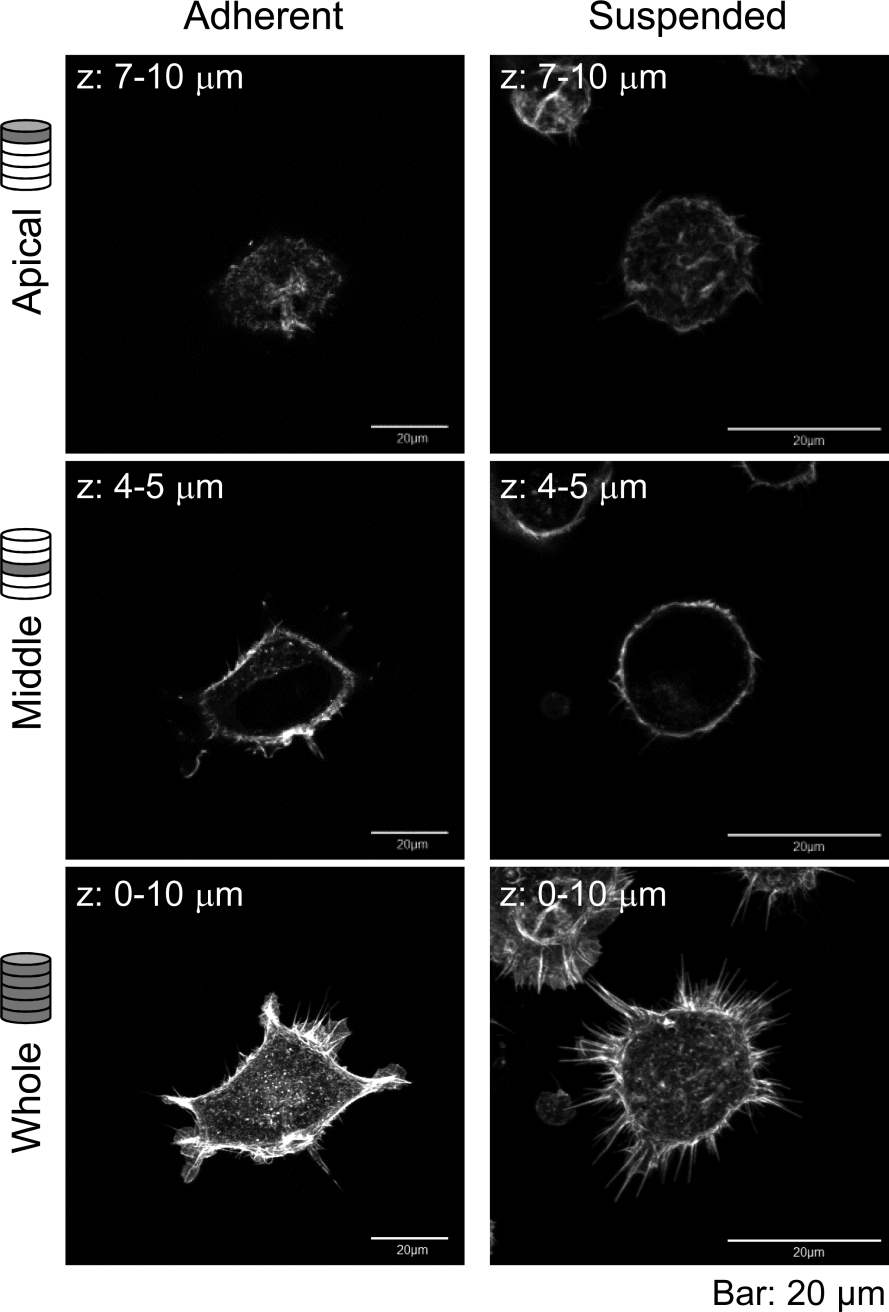
Confocal laser-scanning microscopy (CLSM) images of fluorescently labeled F-actin of adhered and BAM-anchored suspended HEK293 cells. Superimposed images of the apical cell surface (Apical), middle part (Middle), and whole cell (Whole) are shown. The thickness of superimposed images (*z*) is shown individually.

Furthermore, the actin cytoskeleton structures of HEK293 cells cultured under these conditions were observed by CLSM (Fig. 2). Apparently, there was not much difference in F-actin structures of HEK293 cells in the adherent and suspended states. Immature F-actin structures were observed on the apical surface of both spread and spherical cells. The peripheral F-actin structures at the plasma membrane were observed in the middle part of the adherent cells. In suspended cells, a clear ring-shaped cortical actin was visible in the middle part of the serial image. Whole-cell imaging revealed numerous dot-shaped F-actin structures inside the adherent cells, while a lot of projections appeared on the surface of suspended cells. No developed actin stress fibers were noticed in both adhesion states. Thus, with respect to the possible observations of the F-actin structures in HEK293 cells by CLSM, the structures appeared to be immature by nature and seemed to be unchanged after removing the cells from the culture surface.

### Mechanical properties of adherent and BAM-anchored suspended HEK293 cells

We next determined the surface mechanical properties of the cells in the adherent and suspended states using the AFM indentation method. AFM indentation is a sensitive method for analyzing the actin microstructures on the cell surface (Haghparast et al., 2013; Shimizu et al., 2012a). To reduce the influence of cell morphology in each adherent cell, we placed the AFM probe onto the center of the single cell surface.

**Figure 3:**
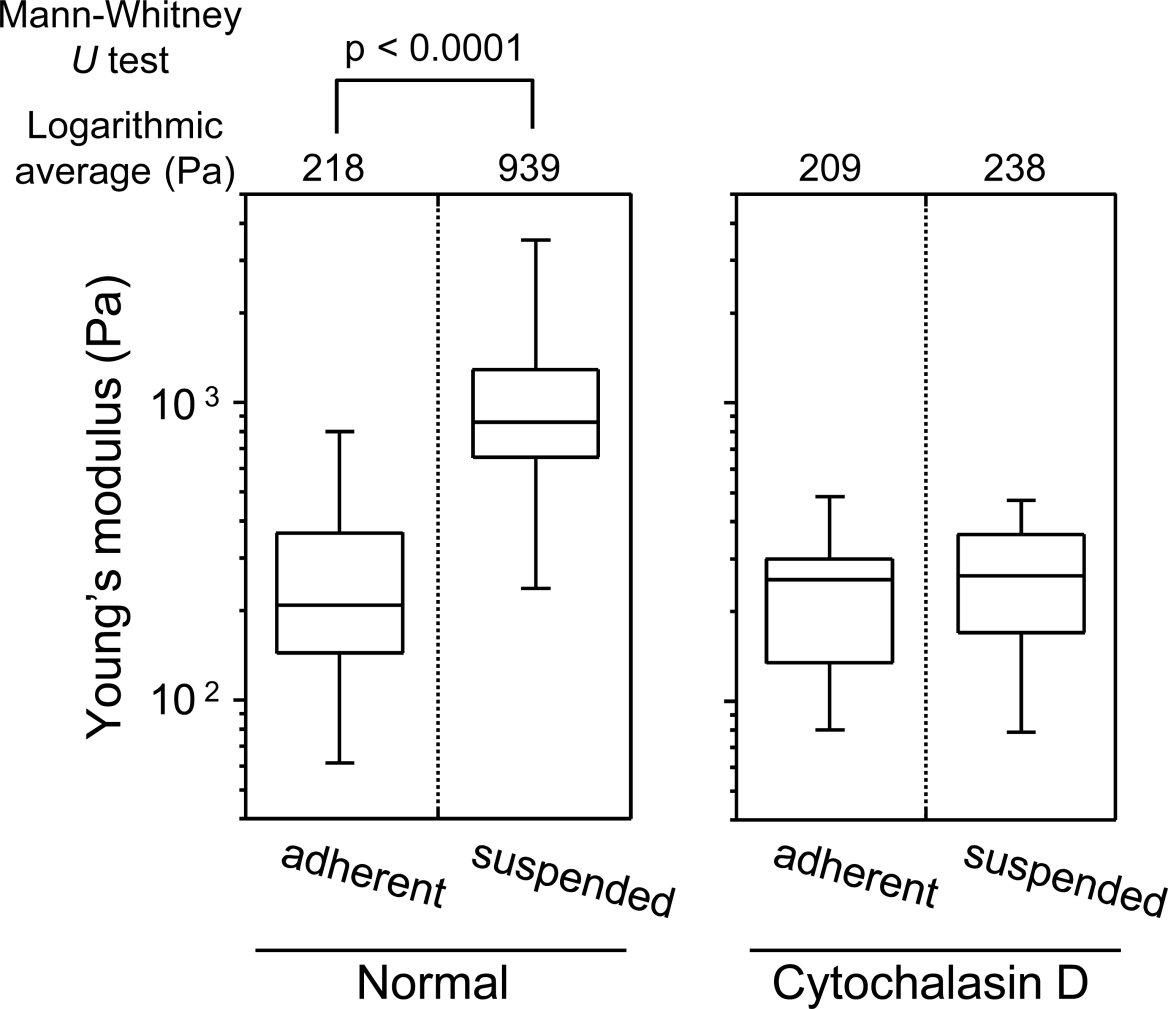
Young’s modulus of HEK293 cells in the adherent and suspended states. The distributions of the Young’s moduli in adherent and suspended states are shown as box-and-whisker plots (Normal). The Young’s moduli of the cells treated with Cytochalasin D are shown on the right (Cytochalasin D). The logarithmic average of the Young’s moduli is shown on the top of each plot. Each condition shows the Young’s modulus of more than 20 individual cells.

Figure 3 shows the distribution of the Young’s modulus of the cells in the two adhesion states. The Young’s modulus values were broadly distributed, irrespective of the cell adhesion state. The distribution of the Young’s moduli of suspended round cells was clearly higher than that of adherent cells (Fig. 3). In short, upon detachment from the substrate and development of a round transformed morphology, distribution of the Young’s modulus of HEK293 cell surface increased.

The sharp drop of the Young’s modulus due to actin depolymerization with cytochalasin D indicates a significant contribution of the actin structures to the elevation of surface stiffness of suspended HEK293 cells (Fig. 3). By contrast, the Young’s modulus of adherent cells was almost unchanged by treatment with cytochalasin D. It indicates that the visually similar cell-surface F-actin structures of HEK293 cells in the adherent and suspended states were fundamentally different (Figs. 2 and 3, Fig. S1).

### Mechanical responsiveness of HEK293 cells to perturbations of the actin cytoskeleton

To evaluate the contribution of F-actin structures to the mechanical properties of HEK293 cells, we examined their responsiveness to the actin cytoskeleton-modifying agents Y27632 and calyculin A. Y27632 is a ROCK inhibitor that prevents stress fiber formation (Uehata et al., 1997). On the other hand, calyculin A is a myosin light chain phosphatase inhibitor that activates actomyosin formation and enhances actin polymerization (Ishihara et al., 1989). F-actin CLSM images of the cells treated with Y27632 and calyculin A are shown in Fig. S1. Although the morphology of the drug-treated adhered cells was changed, the morphology of the drug-treated suspended cells did not change (Fig. S1).

**Figure 4:**
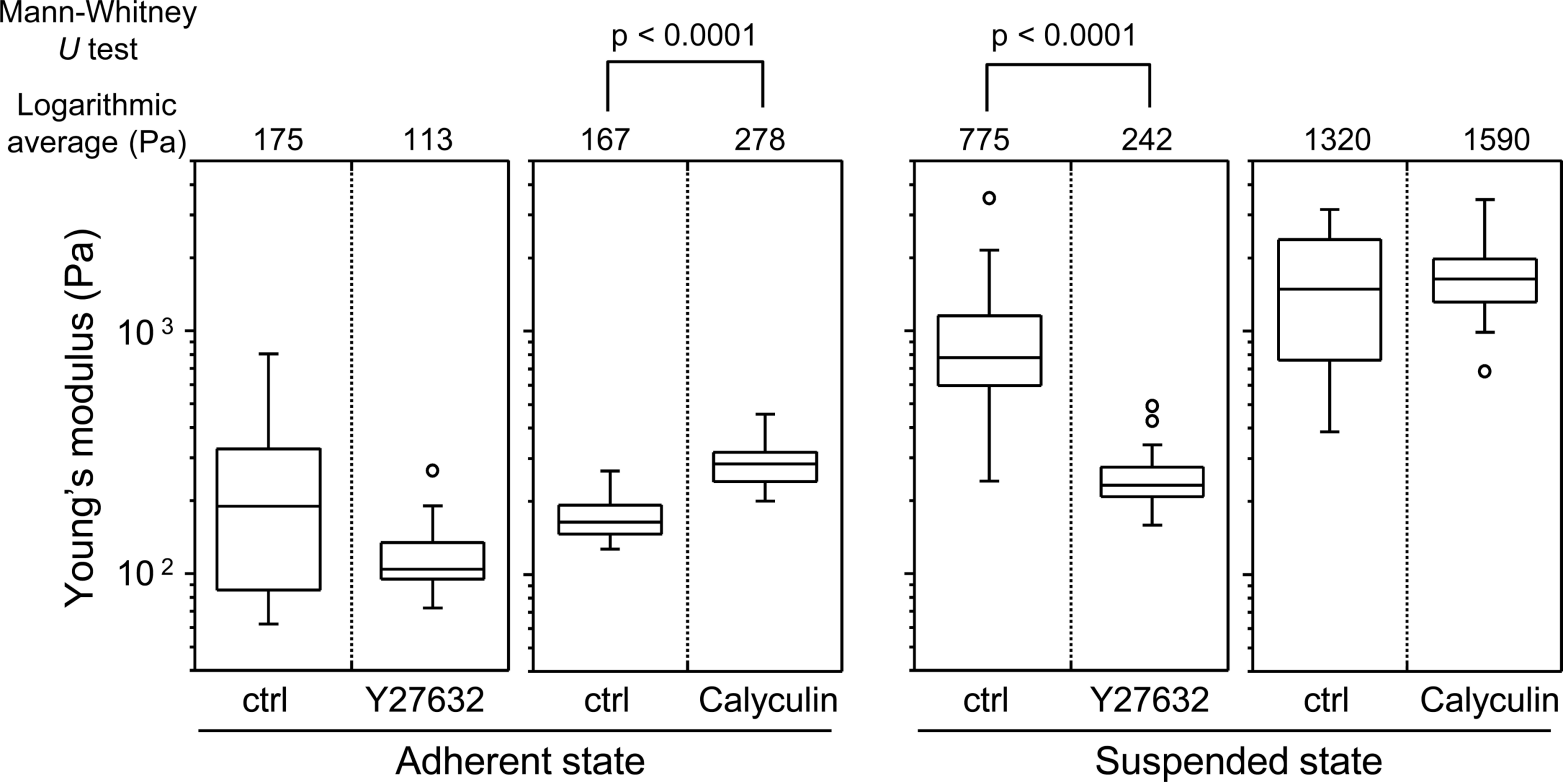
Elastic responses of adherent and BAM-anchored suspended cells following treatment with Y27632 and calyculin A. The distribution and logarithmic average of the Young’s modulus of HEK293 cells in adherent and suspended states are shown. Each condition shows the Young’s modulus of 20 individual cells.

In the adherent state, the Young’s modulus of the cell surface slightly decreased by treatment with Y27632, but increased by treatment with calyculin A (Fig. 4). On the other hand, in the suspended state, the Young’s modulus clearly decreased by addition of Y27632 and was almost unchanged after addition of calyculin A (Fig. 4). Thus, the mechanical responsiveness of surface F-actin of HEK293 cells differs depending on whether the cells are in adherent or suspended state.

**Figure 5:**
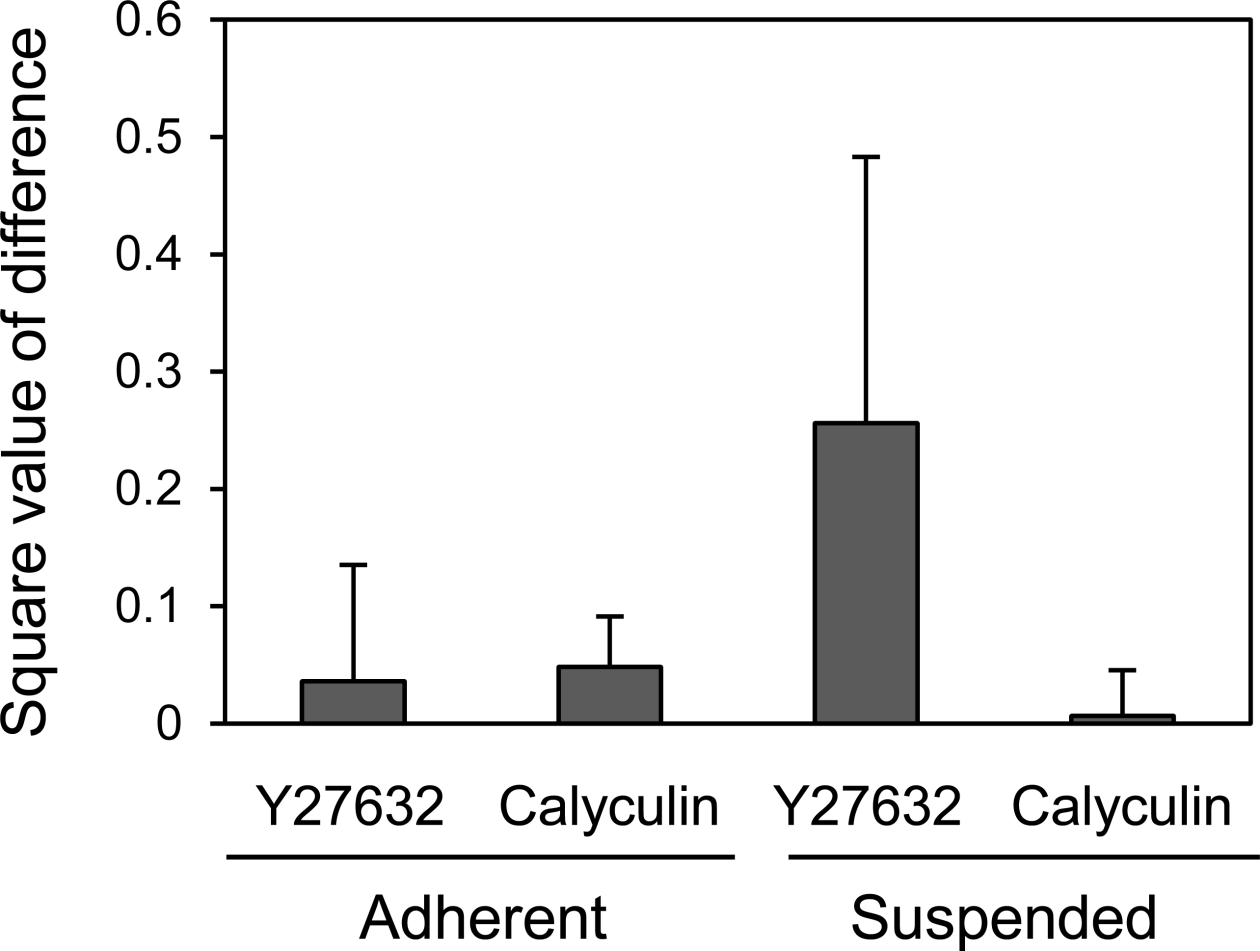
Mechanical response of adherent and BAM-anchored suspended HEK293 cells after treatment with Y27632 and calyculin A. The squared values of the logarithmic average difference of the Young’s modulus in the non-treated control condition versus the agent-treated condition were used for the comparison.

Finally, we compared the mechanical responsiveness of HEK293 cells to each drug in each adhesion state (Fig. 5). The squared values of the changes in the logarithmic average of the Young’s modulus in response to Y27632 and calyculin A were used for the comparison. HEK293 cells were hardly affected by calyculin A and Y27632 in the adherent state. However, in suspended state, they were strongly affected by Y27632, but were not affected by calyculin A. Thus, the mechanical responsiveness of HEK293 cells to actin-modifying agents was clearly different in the adherent and suspended states. Our previous data for normal stromal i.e., mesenchymal stem cells and normal fibroblasts, and cancer cells i.e., HeLa and HT1080 cells, showed that the normal stromal cells were strongly affected by only Y27632 in the adherent and suspended states, whereas the cancer cells were equally affected by calyculin A and Y27632 treatment irrespective of their adhesion state (Haghparast et al., 2013). Therefore, the regulatory mechanisms for F-actin structures of HEK293 cells were different depending on the adhesion state; in the adherent state, the mechanism was similar to that of cancer cells, but in the suspended state, it was similar to that of normal stromal cells (Haghparast et al., 2013). Therefore, the surface F-actin architecture of HEK293 cells varies from that of normal stromal and cancer cells.

## Discussion

This study discerned the surface mechanics and actin cytoskeleton architecture of HEK293 cells in adherent and suspended states. HEK293 cells were generated by viral transformation (Graham et al., 1977) and resemble cancer cells. Thus, we initially considered their surface mechanical features to be similar to cancer cells. However, their mechanical features were distinct from those of previously reported cancer and normal stromal cells (Haghparast et al., 2013). This suggested that we could categorize the cell surface mechanics into 3 types i.e., normal stromal, cancer, and HEK293 cells.

The mechanical features are largely attributable to the actin cytoskeleton (Dai & Sheetz, 1995; Sugitate et al., 2009; Trickey, Vail & Guilak, 2004; Wang, 1998). It is known that the surface stiffness of adherent fibroblasts or normal stromal cells is borne by the actin cap, which is a fibrous actin structure located above the nucleus (Haghparast et al., 2013; Khatau et al., 2009; Kihara et al., 2011). On the other hand, adherent cancer cells do not have a developed actin cap, but do have many short microvilli on their surfaces (Haghparast et al., 2013). Adherent HEK293 cells lacked the actin cap and bore immature peripheral F-actin structures similar to cancer cells (Fig. 2). Furthermore, detachment from the substrate and the consequent round cell morphology did not cause distinct alterations of F-actin structures at the apical cell surface according to the F-actin imaging (Fig. 2), which was also analogous to the cancer cells (Haghparast et al., 2013). Nevertheless, the Young’s modulus of BAM-anchored suspended HEK293 cells clearly increased compared to that of the cells in the adherent state (Fig. 3). By contrast, the Young’s modulus of cancer cells was almost unchanged after cell detachment from the culture dish (Haghparast et al., 2013; Shimizu et al., 2012a). Thus, augmentation of cortical rigidity by cell detachment is considered to be the unique mechanical feature of HEK293 cells. This augmentation was clearly hindered by induced actin depolymerization (Fig. 3). On the other hand, surface stiffness of the adherent HEK293 cells was almost unchanged by actin depolymerization (Figs. 3 and 4). Analysis of the mechanical properties of the cell surface by AFM provides invisible information about the maturation or strength of actin cytoskeleton networks near cell surface (Haghparast et al., 2013; Shimizu et al., 2012a). Therefore, surface actin cytoskeleton of HEK293 cells has undergone a major reorganization and upregulated by cell detachment.

In mitotic *Drosophila* S2R+ cells, cortical rigidity and cell rounding are mainly controlled by ERM proteins, particularly by moesin (Kunda et al., 2008). Moesin helps to convert the protrusive lamellipodial actin structures that dominate in interphase into the uniform cortex characteristic of mitotic cells, in which actin filaments lie parallel to the plane of the plasma membrane (Kunda et al., 2008). Tachibana and colleagues showed that inhibition of integrin-mediated cell adhesion and induction of cell rounding by overexpression of CD43 or CD34 upregulated the phosphorylation of ERM proteins (Ohnishi et al., 2013; Yamane et al., 2011). Furthermore, they reported that detachment of HEK293T cells from the substrate by trypsinization and inhibition of reattachment induced phosphorylation of ERM proteins (Yamane et al., 2011). Thus, the increase of cortical rigidity in trypsinized HEK293 cells observed in the present study could be the result of ERM phosphorylation. In other words, detachment of HEK293 cells induces the phosphorylation of ERM proteins which in turn upregulates the cortical actin, yet further verification studies are required.

In summary, the surface F-actin architecture of HEK293 cells can be categorized into a discrete group that is distinct from that of normal stromal and cancer cells. Adherent HEK293 cells bore immature peripheral F-actin structures leading to very low surface stiffness. On the other hand, detachment from the culture substrate by trypsinization upregulated the surface actin structure, which resulted in augmentation of cell surface stiffness. The elastic responsiveness of HEK293 cells to the actin-modifying agents Y27632 and calyculin A were distinct in the two adhesion states. The surface F-actin of adherent HEK293 cells showed a similar response to the actin-modifying reagents as previously observed for adherent cancer cells. On the other hand, the surface F-actin of suspended HEK293 cells showed a similar response to the actin-modifying reagents as that of suspended normal stromal cells. Thus, we succeeded in identifying a third mechanically distinct cell type in addition to normal stromal and cancer cells. In the future, we would like to construct a database for mechanical features of various cell types and present a new classification system based on cell mechanics.

## Supplemental Information

10.7717/peerj.1131/supp-1 Figure S1 Confocal laser-scanning microscopy images of fluorescently labeled F-actin of adhered and BAM-anchored suspended HEK293 cells treated with actin modifying agents Superimposed images of the whole cell (left) and orthogonal *Y*–*Z* images (right) are shown.

10.7717/peerj.1131/supp-2 Figure S2 Raw data of Young’s modulus of tissue culture polystyrene and BAM-coated surfaces

10.7717/peerj.1131/supp-3 Figure S3 Raw data of Fig. 3
Raw data of Young’s modulus of HEK293 cells on normal culture surface and BAM-coated surface. The cells were treated with or without cytochalasin D.

10.7717/peerj.1131/supp-4 Figure S4 Raw data of Fig. 4
Raw data of Young’s modulus of HEK293 cells after treatment with Y27632 and calyculin A on normal culture surface and BAM-coated surface.
