# Supplementary figures and images for: Distinct mechanical behavior of HEK293 cells in adherent and suspended states
Source: PeerJ. 2015 Jul 30;3:e1131. doi: 10.7717/peerj.1131 (PMC4525692; doi:10.7717/peerj.1131)

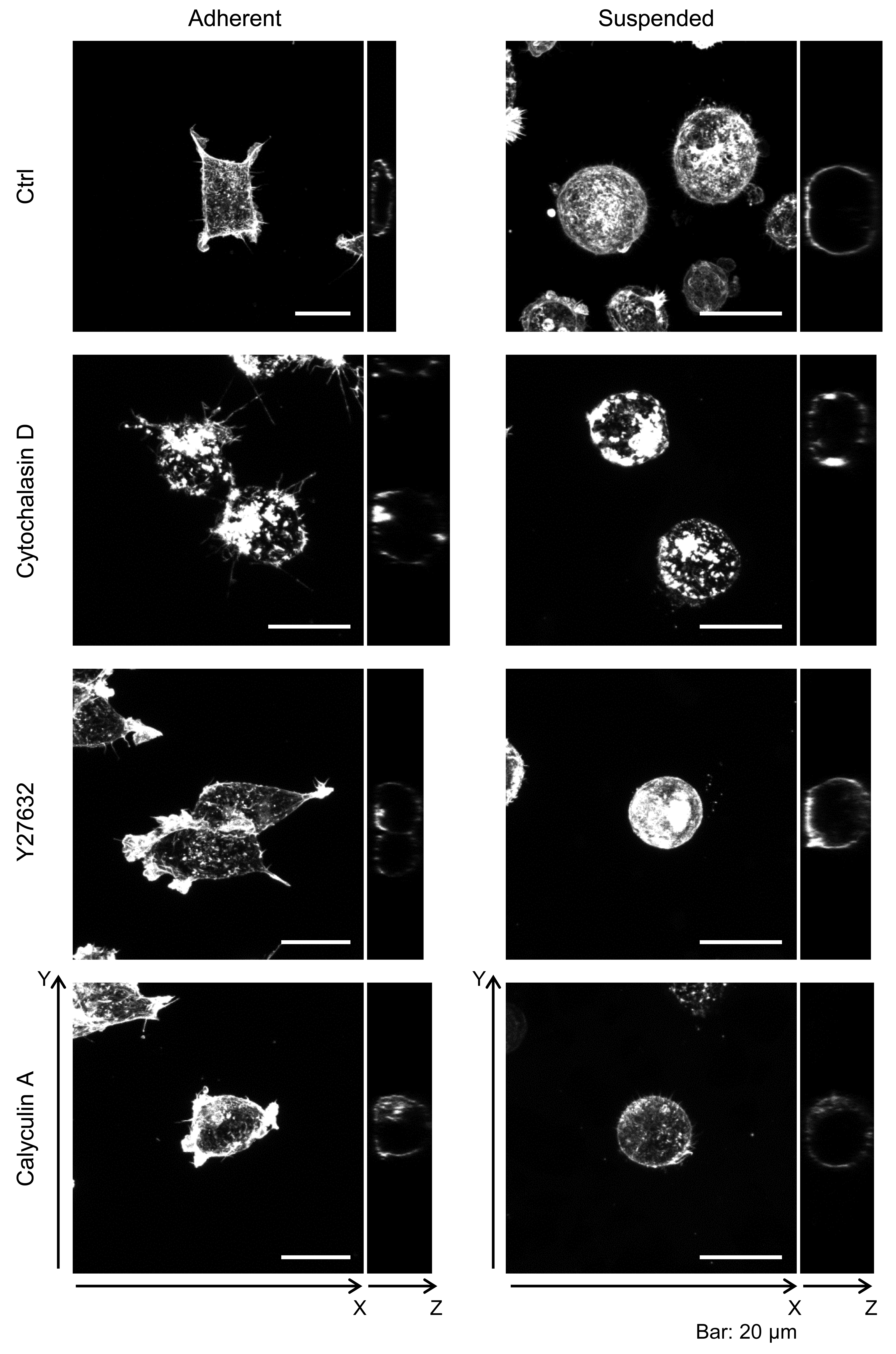

Supplement: Figure S1 — Superimposed images of the whole cell (left) and orthogonal Y–Z images (right) are shown. [file peerj-03-1131-s001.png]
